# Response to: Once-nightly sodium oxybate (FT218) in the treatment of narcolepsy: a letter to the editor commenting on the recent publication by C. Kushida et al

**DOI:** 10.1093/sleep/zsac042

**Published:** 2022-06-13

**Authors:** Clete A Kushida, Thomas Roth, Colin M Shapiro, Asim Roy, Russell Rosenberg, Akinyemi O Ajayi, David Seiden, Jennifer Gudeman

**Affiliations:** Department of Psychiatry and Behavioral Sciences, Stanford University Medical Center, Palo Alto, CA, USA; Sleep Disorders and Research Center, Henry Ford Health System, Detroit, MI, USA; University of Toronto, Toronto, ON, Canada; Ohio Sleep Medicine and Neuroscience Institute, Dublin, OH, USA; Neurotrials Research, Inc., Atlanta, GA, USA; Florida Pediatric Research Institute, Winter Park, FL, USA; Avadel Pharmaceuticals, Chesterfield, MO, USA; Avadel Pharmaceuticals, Chesterfield, MO, USA

Dear Editor,

We thank F. Skobieranda, S. Candler, and W. Macfadden from Jazz Pharmaceuticals, Inc. for their comment on “Once-Nightly Sodium Oxybate (FT218) Demonstrated Improvement of Symptoms in a Phase 3 Randomized Clinical Trial in Patients With Narcolepsy.” We appreciate their recognition of how this research will expand the legacy of sodium oxybate, which is the established gold standard in the treatment of narcolepsy.

Skobieranda et al. comment on the study design and discussion of efficacy and safety of FT218, or ON-SXB, as well as other oxybate formulations. REST-ON was conducted under a Special Protocol Agreement (SPA) assessment with the Food and Drug Administration (FDA), which is reached when the FDA agrees with the “adequacy and acceptability of specific critical elements of overall protocol design (e.g., entry criteria, dose selection, endpoints, and planned analyses)” allowing the study to be considered adequate and well-controlled to support a New Drug Application [1]. The ON-SXB trial design used a placebo-controlled evaluation of three therapeutic doses (6, 7.5, and 9 g) of ON-SXB, with evaluation at three prespecified endpoints: week 3 for 6 g, week 8 for 7.5 g, and week 13 for 9 g [2], similar to the dose escalation used in clinical practice.

The second comment is about the discussion of efficacy and tolerability rates. As noted in the publication, the discussion section explicitly stated that cross-trial comparisons should be interpreted with caution owing to different trial designs [2]. Given that clinicians and their patients may have a choice of 3 oxybate therapies if ON-SXB is FDA approved, the recognition of efficacy and safety data generated through these separate trials is relevant. Skobieranda et al. attribute the lack of efficacy observed with the 6-g dose of immediate-release, twice-nightly sodium oxybate (Xyrem) [3], in part to the shorter duration (4 weeks compared to 13 weeks). As described in the REST-ON trial design, the evaluation of ON-SXB 6 g occurred at week 3 [2].

The third comment focused on the relative tolerability of once-nightly vs twice-nightly oxybate. The rates cited for ON-SXB were also treatment-emergent adverse events, not adverse reactions. Furthermore, the cited treatment-emergent adverse events were obtained from the published Jazz literature, in which events with a p value of <.05 were considered related [3].

The letter authors contest the notion that falls related to the middle-of-the-night dosing may potentially be lessened with a single bedtime dose and remind readers that the Xyrem and Xywav Prescribing Information advise patients to stay in bed after the second dose [4, 5], guidance that was added to the labeling in 2014 based on postmarketing surveillance reports. Falls may occur with any oxybate formulation. If ON-SXB is FDA approved, clinical practice will likely elucidate whether a single bedtime dose mitigates this risk.

The final comment focuses on the lower sodium included in the recently introduced mixed-salt oxybates. Sodium oxybate, which contains approximately 1600 mg of sodium at its highest dose, has been used in the United States for nearly 20 years and in Europe for more than 15 years, providing a robust dataset to analyze for potential correlation to cardiovascular disease. When a comprehensive review of the literature was undertaken, no association between sodium oxybate use and cardiovascular disease was identified [6]. Beyond these reassuring findings, debate exists over the appropriate recommended threshold for sodium intake; the European Society of Cardiology has asserted that a population-level mean target below 5,000 mg per day is reasonable [7].

Sodium oxybate remains underutilized 2 decades later after its introduction in 2002. We realize that innovation in medicine is often incrementally achieved, evolving the status quo. It is our sincere hope that if ON-SXB is approved by the FDA, the 3 oxybate formulations will allow clinicians to have a more patient-centered, personalized medicine approach to narcolepsy care by enabling them to prescribe the oxybate medication that best meets their patients’ individual needs.

## Data Availability

All relevant data are included within the text.
